# CYP2D6 genotypes, endoxifen levels, and disease recurrence in 224 Filipino and Vietnamese women receiving adjuvant tamoxifen for operable breast cancer

**DOI:** 10.1186/2193-1801-2-52

**Published:** 2013-02-15

**Authors:** Richard R Love, Zuerenesay Desta, David Flockhart, Todd Skaar, Evan T Ogburn, Anuradha Ramamoorthy, Gemma B Uy, Adriano V Laudico, Nguyen Van Dinh, Le Hong Quang, Ta Van To, Gregory S Young, Erinn Hade, David Jarjoura

**Affiliations:** International Breast Cancer Research Foundation, 505 S Rosa Rd Ste 35E, Madison, WI 53719 USA

## Abstract

**Background:**

While tamoxifen activity is mainly due to endoxifen and the concentration of this active metabolite is, in part, controlled by CYP2D6 metabolic status, clinical correlative studies have produced mixed results.

**Findings:**

In an exploratory study, we determined the CYP2D6 metabolic status and plasma concentrations of endoxifen among 224 Filipino and Vietnamese women participating in a clinical trial of adjuvant hormonal therapy for operable breast cancer. We further conducted a nested-case–control study among 48 women (half with recurrent disease, half without) investigating the relationship of endoxifen concentrations and recurrence of disease.

We found a significant association of reduced endoxifen plasma concentrations with functionally important CYP2D6 genotypes. High endoxifen concentrations were associated with higher risk of recurrence; with a quadratic trend fitted to a stratified Cox proportional hazards regression model, the likelihood ratio p-value was 0.002. The trend also showed that in 8 out of 9 pairs with low endoxifen concentrations, the recurrent case had lower endoxifen levels than the matched control.

**Conclusions:**

This exploratory analysis suggests that there is an optimal range for endoxifen concentrations to achieve favorable effects as adjuvant therapy. In particular, at higher concentrations (>70 ng.ml), endoxifen may promote recurrence.

**Electronic supplementary material:**

The online version of this article (doi:10.1186/2193-1801-2-52) contains supplementary material, which is available to authorized users.

## Introduction

Although *in vitro* and *in vivo* studies have strongly suggested that tamoxifen activity is mainly due to endoxifen and that the concentration of this active metabolite is in part controlled by CYP2D6 metabolic status, clinical correlative studies have produced mixed results. The lack of consistent results may be in part due to the retrospective nature of most of the clinical studies. In addition, endoxifen plasma concentrations are highly variable among patients. While CYP2D6 is the main enzyme catalyzing the formation of endoxifen from N-desmethyltamoxifen, CYP2D6 metabolic status only accounts for 10-19% of the variability (Desta et al. 2004; Jin et al. 2005). Thus, over 80% of endoxifen inter-patient variability remains unexplained by CYP2D6. In light of the possibility that multiple pathways control endoxifen plasma concentrations, and given that the pharmacogenetic studies so far reported are not consistent, it is likely that the absolute endoxifen concentrations better predict tamoxifen activity in patients. The multiplicity of studies available evaluating pharmacogenomic hypotheses with tamoxifen have investigated CYP2D6 genotypes and outcomes, and these considerations likely explain why these reports do not provide a clear picture of the relationship (Dezentjé et al. 2009; Hayes et al. 2008; Kiyotani et al. 2010; Lash and Rosenberg 2010).

We hypothesize that endoxifen concentration, rather than a single gene variation, may be a better biomarker of tamoxifen response.

In the context of a clinical trial where all patients were receiving tamoxifen as part of their adjuvant therapy for hormone receptor-positive breast cancer, here we report preliminary data addressing associations of disease recurrence with CYP2D6 genotypes and endoxifen plasma concentrations, in the first 224 Asian subjects we have studied in the trial.

## Methods and patients

### Patients

Provisional IRB approval was first obtained at the principal investigator’s (RRL) American institution following which specific approval was obtained from in-country appropriate IRBs in the Philippines and Vietnam, and at co-investigators’ institution (Indiana University), again following which the principal investigator’s institution provided final approval. The parent clinical trial whose participants were recruited was similarly approved by the foreign and principal investigator’s IRBs.

This ancillary study was begun after the parent clinical trial had accrued 40% of subjects (total accrual =740). Some patients on the parent trial refused participation in the current ancillary study. Thus the patients entering the current study are not representative of all patients entering the parent trial. The parent trial is a randomized clinical trial evaluating the impact of the timing of surgical oophorectomy in the menstrual cycle on outcomes from operable, hormone receptor positive breast cancer. All patients in the trial underwent surgical oophorectomy on the same day as their primary breast surgery, and began tamoxifen 20 mg per day within five days after this.

We have reasons to believe that the compliance with tamoxifen administration (Nolvadex, supplied by AstraZeneca) is high and thus this factor seems less likely to affect the current pharmacogenetic studies. Specifically, patients are expected to return at 3-month intervals to get their drug, which is free. We have logs recording patient collection of tamoxifen medication. The tamoxifen, endoxifen and other metabolite levels in the 224 individual patients studied to date (*vide infra*) are all such that regular tamoxifen consumption must be occurring. Patients are not taking any drugs believed to interfere with tamoxifen metabolism which issue has confounded reported studies in western populations.

Plasma, leukocyte and serum specimens after four or more months of tamoxifen treatment were obtained in light-shielded tubes from 224 Vietnamese and Filipino patients. The specimens were frozen at −70 degrees C, and shipped frozen in liquid nitrogen canisters to the Indiana University co-investigators. The logistics of sample collection, storage and transport from these foreign countries to the US have been carefully conducted and are validated by the preliminary data we generated. By four months after initiation of tamoxifen, steady-state concentrations of tamoxifen and metabolites are achieved (Jin et al. 2005). Given that the volume of distribution of tamoxifen and metabolites is large, with long half-lives, diurnal and day-to-day variation in these concentrations in an individual patient is minimal (Lash and Rosenberg 2010).

### Laboratory methods

The CYP2D6 gene is highly polymorphic, with over 70 alleles that exhibit functional consequences identified. We have genotyped for CYP2D6 variants relevant to Filipino and Vietnamese populations.

Genomic DNA was isolated from the leukocytes using the QIAamp DNA Blood Maxi Kit (Qiagen, Valencia, CA, USA) and stored at −20°C until use. The DNA concentrations were measured by Quant-iT DNA Broad Range Kit (Invitrogen, Carlsbad, CA, USA). The DNA was diluted to10 ng/μl and used for performing the CYP2D6 genotyping assays. The genotyping was performed for CYP2D6 alleles *2, *3, *4, *5, *6, *10, and *41 using the predeveloped TaqMan Genotyping Assays (Applied Biosystems, Foster City, CA, USA) following the manufacturer’s instructions. CYP2D6 is a highly polymorphic gene with more than 70 different allelic variants. We chose these star alleles because they are more frequent in the Asian and Caucasian population (Bradford 2002; Sistonen et al. 2007; Veiga et al. 2009).

Tamoxifen and its metabolites were measured by liquid chromatography tandem mass spectrometry (LC-MS/MS) as described elsewhere (Irvin et al. 2011). Standard curves and quality controls were run with each sample batch. The intraday coefficient of variance was lower than 10% for all compounds, while the inter-day was lower than 15%. An intra-patient assay was not run for each sample set, but repeated measurement was performed in a subset of samples. The results show that the intra-sample variability was lower than 10%.

### Statistical methods

We have used our preliminary data to explore the relationship between endoxifen concentrations and recurrence prior to follow-up being complete on all patients in our adjuvant trial. In this effort, we designed a nested case–control study. Our rationale was that using such a small case–control training data set might allow us to validate any important trend in a larger, independent data set collected from our trial patients who will have longer follow-up time in the future (Pepe et al. 2008). Among the 224 studied patients we identified 24 patients who had recurrence, and matched them with 24 non-recurrence controls who were from the same enrollment hospital, were of the same disease stage (I-II vs. III), and had a similar enrollment date. An optimal matching technique was utilized, as implemented by Bergstrahl (Bergstralh et al. 1996) and the follow-up time for the control in each pair was required to be at least as long as the time to recurrence for the case.

The effect of CYP2D6 genotype metabolic status on each of the metabolites (tamoxifen, NDMTAM, endoxifen, and 4-OHTAM) was assessed using a test for linear trend. The proportion of variance explained by the linear trend model (r-squared) was also calculated.

## Results

CYP2D6 allelic frequencies in the two populations were in Hardy-Weinberg Equilibrium except for *1 and *2 in Filipinos (Table 1). Frequencies of specific genotypes are presented in Table 2. On the basis of the genotype-predicted phenotype, genotypes were categorized as normal (fully functional CYP2D6 alleles: *1 and *2), intermediate (alleles associated with reduced enzyme activity: heterozygous for *10 and *41), and slow (homozygous for *10, *41 variants and one or more non-functional null alleles: *3-*6).

**Table 1:** **CYP2D6 allele frequencies in 224 Vietnamese and Filipino patients**

	Frequency (%)	
Allele	Vietnamese	Filipino
*10	58.5	52.4
*1	24	30.0*
*2	11	12.4*
*5	5	2.4
*41	1	2.8
*4	0.5	0
*3	0	0
*6	0	0

^*^CYP2D6 alleles not in Hardy Weinberg equilibrium.

**Table 2:** **CYP2D6 genotypes**

CYP2D6 genotype	Filipino #% of total	Vietnamese #% of total	Total
*1/*1	2	6	8
	1.45	6.98	
*1/*10	66	28	94
	47.83	32.56	
*1/*2	12	3	15
	8.7	3.49	
*1/*41	1	0	1
	0.72	0	
*1/*5	0	3	3
	0	3.49	
*10/*10	37	27	64
	26.81	31.4	
*10/*41	4	1	5
	2.9	1.16	
*2/*10	0	12	12
	0	13.95	
*2/*2	10	1	11
	7.25	1.16	
*2/*5	1	1	2
	0.72	1.16	
*5/*10	5	4	9
	3.62	4.65	
Total cases	138	86	224

**Figure 1:**
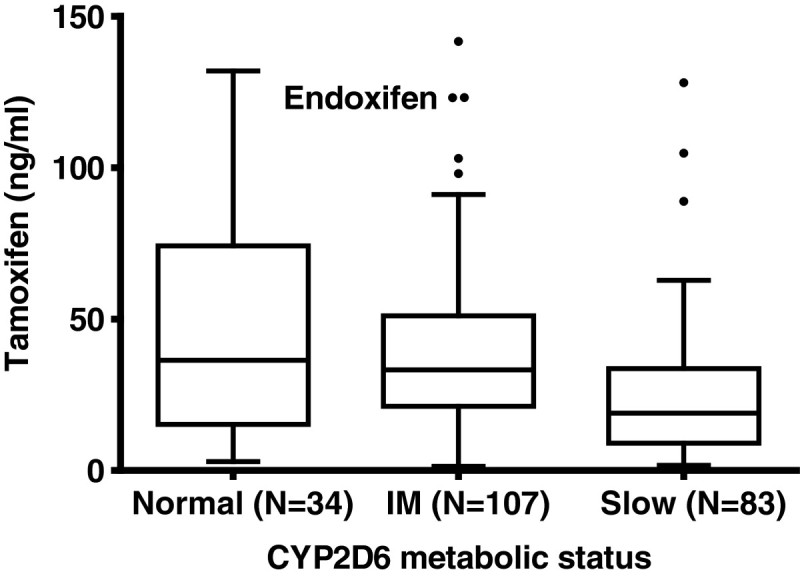
**Endoxifen plasma concentrations by CYP2D6 genotype metabolic status in Filipino (n = 138) and Vietnamese (n = 86) patients (p = 0.0001 for trend: normal > intermediate > slow).**

The data, presented in Figure 1 and Table 3, show wide inter-individual variability in endoxifen concentrations. There was a significant association of reduced endoxifen plasma concentrations with functionally important CYP2D6 genotypes.

**Table 3:** Tamoxifen and metabolites concentrations* in patients with CYP2D6 in 224 Filipino and Vietnamese patients

		CYP2D6 Genotype			Gene-dose effect
Tamoxifen and Metabolites	All (N = 224)	Normal (N = 34)	Intermediate (N = 107)	Slow (N = 83)	Variance explained (p-value for trend)
Tamoxifen (TAM)	195.3 ± 240.9	207.7 ± 256.7	167.7 ± 150.5	226.1 ± 316.2	0.003 (0.39)
NDMTAM	353.3 ± 409.0	325.5 ± 371.1	297.0 ± 238.5	437.2 ± 560.6	0.016 (0.060)
Endoxifen	35.4 ± 28.7	48.2 ± 39.7	39.3 ± 26.6	25.0 ± 22.3	0.086 (<0.0001)
4-OHTAM	1.9 ± 1.5	2.2 ± 1.8	1.9 ± 1.2	1.7 ± 1.7	0.010 (0.13)

*Concentrations (+/− S.D.) are all in ng/ml.

In these data only 10% of the variance in endoxifen concentrations was explained by CYP2D6 genotype. CYP2D6 genotypes were also associated with concentrations of 4-hydroxytamoxifen, another active metabolite of tamoxifen, although this association was less strong, consistent with the partial role CYP2D6 plays in its formation.

### Association of Endoxifen with disease recurrence

A trend in the data for the 48 patients in the nested case–control study showed an unexpected result: high endoxifen concentrations were associated with higher risk of recurrence. We expected that low endoxifen concentrations (below therapeutic values) might increase the hazard of recurrence. Instead, we found that the greatest increase in risk was associated with high endoxifen concentrations (above 70 ng/ml) and that 25% of recurrences had such high concentrations. A quadratic trend fitted to a Cox proportional hazards regression model stratified by matched set demonstrated a likelihood ratio p-value of 0.002. The quadratic variable was created by mean deviating endoxifen concentrations and squaring the deviation. Because the endoxifen concentrations were skewed, the high endoxifen values gave the highest values for this variable, but low endoxifen levels also gave values higher than mean levels, hence the label quadratic effect.

**Figure 2:**
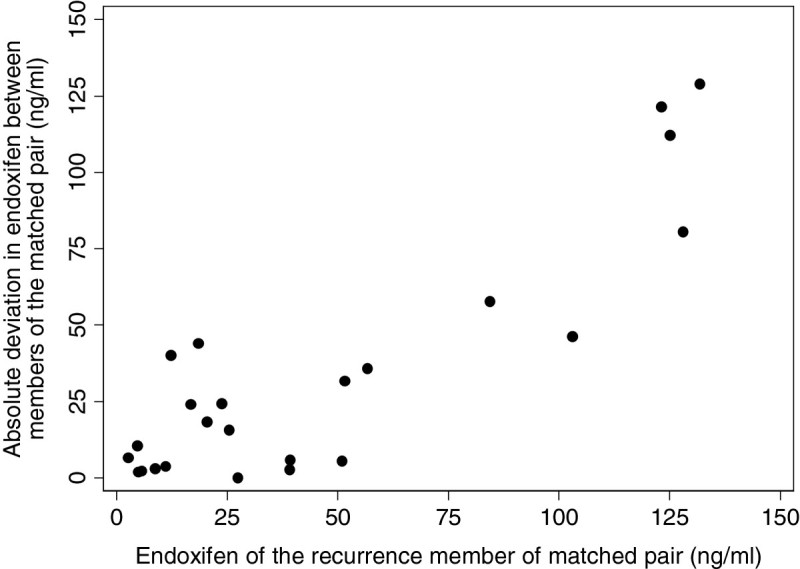
**Plot of the absolute difference in endoxifen levels between members of each matched pair by the endoxifen level of the recurrence member of the matched pair.**

Figure 2 shows the raw data for the 24 matched pairs. It substantiates the significant quadratic effect found in the Cox model. The *x*-axis is the endoxifen concentration of the recurrence member of the pair, and the *y*-axis shows the absolute deviation between endoxifen concentrations of the two members of each pair. For the recurrences with high endoxifen concentrations, the absolute deviations are all large, indicating that the matched controls all had much lower endoxifen levels. The trend also showed that when a recurrence had very low endoxifen levels (below 20), 8 of 9 matched controls had higher values (suggesting a wide J-shaped relationship).

## Discussion

The current report is of an exploratory, hypothesis-generating study. The data here relating CYP2D6 genotypes and endoxifen concentrations are consistent with previous reports with respect to genotype associations, inter-patient variability, and percentage of the inter-patient variability explained by CYP2D6 metabolic status. What is novel and provocative are the data suggesting a J-shaped relationship between endoxifen concentrations in individual patients and likelihood of recurrence. That the suggested increased risks of recurrence at low and high (>70 ng/ml) endoxifen concentrations represent findings which may be validated in the future are supported by:

i. In the treatment of patients with metastatic breast cancer, it has been regularly observed that patients often exhibit a disease “flare” in the first several days after beginning tamoxifen, suggesting that low endoxifen concentrations are estrogenic or disease growth- stimulating.
ii. The incomplete data on the issue of relationships of blood concentrations of endoxifen and effects. The challenges have been to assess concentrations at the effector sites.
iii. The uncertain effects of high-affinity species. We suggest that for the high affinity endoxifen, the wide ranges of blood levels may be associated with remarkably different effects at the receptor sites.
iv. The general observation that despite a strong predictive relationship between tumoral hormone receptor status and response to tamoxifen, in even the best of circumstances responses rates are 60% only and some patients clearly have immediate progressive disease in the face of this therapy.

It is estimated that among “normal” CYP2D6 genoptype carriers, 32% develop steady state endoxifen levels of >70 ng/ml with tamoxifen 20 mg/day (range 71–142 ng/ml) (Borges et al. 2006). Our observation therefore, if confirmed, will carry significant relevance to the issue of optimizing use of this treatment worldwide. In addition, our findings may have important implications as oral endoxifen is under clinical investigation as a new treatment for breast cancer (Ahamed et al. 2010).

## Authors’ information

From The International Breast Cancer Research Foundation, Madison, WI (RRL), Indiana University, Indianapolis, IN (ZD, DF, TS, ETO, AR), Philippine General Hospital, Manila, PH (GBU, AVL), Hospital K, Hanoi Vietnam (NVD, LHQ, TVT) and The Ohio State University, Columbus, OH (GSY, EH, DJ)

Supported in part by NIH RO1 CA 097375, The Breast Cancer Research Foundation, and The International Breast Cancer Research Foundation

## Authors’ original submitted files for images

Below are the links to the authors’ original submitted files for images. Authors’ original file for figure 1 Authors’ original file for figure 2
