# Supplementary figures and images for: CYP2D6 genotypes, endoxifen levels, and disease recurrence in 224 Filipino and Vietnamese women receiving adjuvant tamoxifen for operable breast cancer
Source: Springerplus. 2013 Feb 15;2(1):52. doi: 10.1186/2193-1801-2-52 (PMC3584248; doi:10.1186/2193-1801-2-52)

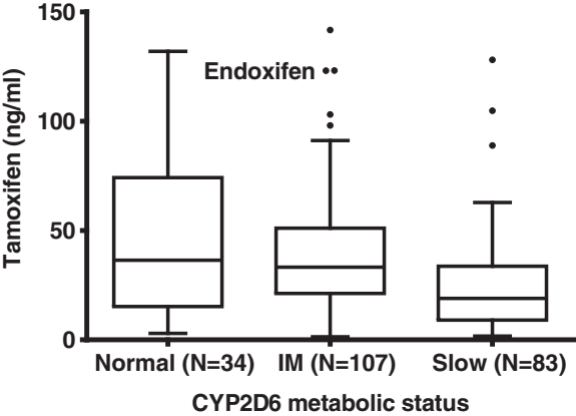

Supplement: Supplementary file 1 — Authors’ original file for figure 1 [file 40064_2013_97_MOESM1_ESM.pdf]

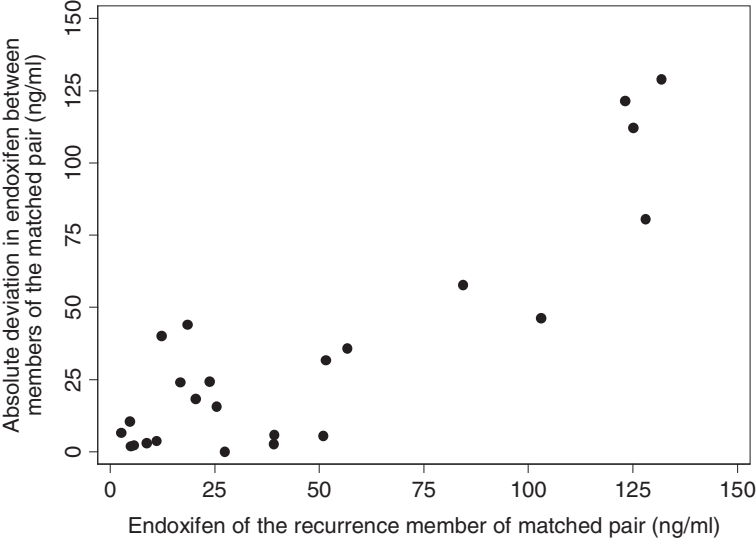

Supplement: Supplementary file 2 — Authors’ original file for figure 2 [file 40064_2013_97_MOESM2_ESM.pdf]
